# Safety of anti-TNF therapy: 10 year experience in pediatric patients from a single tertiary center

**DOI:** 10.1186/1546-0096-9-S1-P68

**Published:** 2011-09-14

**Authors:** G Espada, SM Meiorin, L Guerini, M Alvarez, Giupponi

**Affiliations:** 1 Rheumatology Section Hospital de Niños”Dr Ricardo Gutierrez”, Buenos Aires, Argentina

## Background

Anti-TNF agents are frequently used in the treatment of severe rheumatic disorders in childhood mainly Juvenile Idiopathic arthritis. Despite good clinical efficacy of this therapy, practitioners must remain alert for potential side effects, particularly after prolonged use (infections, malignancy and autoinmune events) .

## Objective

Anti-TNF (Etanercept, infliximad and adalimumab) related adverse events were consecutively recorded and analized in pediatric patients with rheumatic disorders followed in our section from 2000 to 2010.

## Methods

Time of exposure, dose and reason for discontinuation were recorded Analysis involved incidence rate, outcome, and causal relationship. Statiscal analysis were performed using SPSS software.

## Results

Data from 131 clinical records were analyzed . 87(66.4%) were female, median age at diagnosis 7.2 (RIQ 3.8-11.3), median follow up 7.9 ys (SD±4.8). 111 pts had JIA,5 JDM ,4 vasculitis, 3 sarcoidosis, 2MCTD,1SLE and 5 idiopathic uveitis.

**Table:** 

	Etanercept	Infliximab	Adalimumab
Number of treatments	105	43	12
Mean age at onset,ys (SD)	11.2 (4.8)	12.5 (4.1)	16.4 (3.8)
Exposure time (pts-year)	246,5	82,7	9,2
Adverse events	75	76	5
Incidente rate AE	0.3	0.9	0.5
SAE (incidente rate)	2 (0.008)	5 (0.06)	0 (0)
Opportunistic infections (Tuberculosis)	1	2	0
Discontinuation due to AE n (%)	5 (4.8)	13 (30.2)	0

Most of the AEs were mild: 28,2% and 13,5 upper respiratory and skin infections respectively.

Four pts developed autoinmune events: Lupus –like syndrome, Psoriasis and 2 other ,optic neuritis .One pt under IFX + MTX developed Non-Hodgkin Limphoma and another on IFX fatal fulminant hepatitis.

## Conclusions

In our series we observed a total of 156 AEs with incidence rates of: 0.3, 0.5 and 0.9 with Etanercept,adalimumab and infliximab respectively.

Seven pts developed SAEs including opportunistic infections (TB), serious infusion reactions ,NHL and fulminant hepatitis. Eigtheen pts (13,8 %) discontinued antiTNF therapy due adverse events.

